# Clinical and laboratorial characterization of a cohort of patients with hereditary platelet disorders in Brazil

**DOI:** 10.1016/j.htct.2025.103837

**Published:** 2025-04-28

**Authors:** Letícia Dalla Vecchia Grassi, Erica Okazaki, Cynthia Rothschild, Paula Villaça, Fernanda Andrade Orsi, Bianca Stefanello

**Affiliations:** a Hospital das Clínicas, Faculty of Medicine, University of São Paulo, São Paulo, São Paulo, Brazil; b School of Medical Sciences, University of Campinas, Campinas, São Paulo, Brazil

**Keywords:** Thrombocytopenia, inherited coagulation disorders, platelets, epidemiology, diagnosis

## Abstract

**Introduction:**

Inherited platelet disorders are rare conditions characterized by altered platelet function and/or reduced platelet counts. Diagnosing these disorders is challenging and may result in delays, misdiagnosis, and inappropriate treatment. In low- and middle-income countries, data are scarce. Here, we describe a cohort of patients at a reference center in Brazil.

**Methods:**

A descriptive analysis was conducted on patients followed at the Thrombosis and Hemostasis outpatient clinic of the Hospital das Clinicas, University of São Paulo, Brazil.Medical records of 857 patients with thrombocytopenia or bleeding disorders of unknown cause, evaluated between 1998 and 2023, were reviewed. Of these, 60 patients had a confirmed or suspected diagnosis of an inherited platelet disorder and were included in the study.

**Results:**

Among the 60 patients, the majority were female (75 %), with a median age of 48 years. The suspicion of a platelet disorder was based on clinical presentation, family history, and laboratory findings. Overall, 65 % of the patients had abnormal platelet function, while 35 % presented with thrombocytopenia. A positive family history was reported in 62 % of those with low platelet counts and in 51 % of patients with platelet function abnormalities. Previous misdiagnoses included immune thrombocytopenia and von Willebrand disease. Overall, the bleeding phenotype was mild, with a median ISTH-BAT (International Society on Thrombosis and Haemostasis Bleeding Assessment Tool) score of 6. Patients with reduced platelet counts tended to have lower ISTH-BAT score.

**Conclusions:**

Identifying inherited platelet disorders is essential for proper treatment and follow-up. This study emphasizes the need for careful assessment of family history, bleeding risk, platelet count, morphology, and function for diagnosis, particularly in low-resource settings without access to advanced genetic testing.

## Introduction

Inherited platelet disorders (IPDs) encompass a diverse range of conditions characterized by altered platelet function (inherited platelet function disorders - IPFDs) and reduced platelet counts (inherited platelet number disorders - IPNDs).^1^, ^2^, ^3^ These diseases are rare, with an estimated prevalence of 2–3 cases per 100,000 people.^3^^,^^4^ Clinical presentations vary widely, with patients experiencing mild to severe bleeding episodes, sometimes accompanied by other findings such as decreased platelet counts, abnormal platelet morphology, syndromic features, or a predisposition to other systemic diseases.^5^ Their pathophysiology is still being investigated. With advancing genetic mapping tools, many new entities are being identified. Today, 60 distinct IPDs and 75 related genes are recognized.^5^^,^^6^

Diagnosing IPDs is challenging and often leads to delays or misdiagnosis. Diagnostic assessments involve evaluating the location, triggers, severity of bleeding episodes, concurrent syndromic features, and relevant family history. Initial evaluations include examining platelet count and morphology. Additional tests, such as platelet aggregation, immunophenotyping, and electron microscopy, may be utilized to assess platelet function. Genetic mapping can aid in diagnosis by identifying mutations associated with inherited platelet disorders though its accessibility is often limited.^7^^,^^8^

Lack of knowledge about IPDs, combined with the heterogeneity of clinical manifestations and the difficulty of laboratory confirmation, can lead to delays in diagnosis and subsequently in treatment. Accurately identifying and characterizing these disorders is crucial for providing appropriate treatment, genetic counseling, and follow-up, especially considering their association with systemic diseases and neoplasias.^7^^,^^8^

Most of the existing data on these diseases is derived from registries in North America and Europe. In low- and middle-income countries, such as Brazil, there is a lack of data describing the characteristics of the population and the challenges faced in diagnosing this group of diseases, particularly in the context of the public health system with its limited resources.

In this context, this study describes a cohort of patients with IPDs followed at a reference center in Brazil. The objective is to contribute to the limited existing body of evidence on the clinical presentation of these diseases in low- and middle-income countries, with the ultimate goal of increasing awareness of IPDs and their diagnosis in these countries.

## Methods

A retrospective descriptive analysis was conducted of a cohort of patients with suspected or confirmed IPD followed at the Thrombosis and Hemostasis outpatient clinic of the Hospital das Clinicas, Faculty of Medicine, University of São Paulo (HCFMUSP), Brazil.

The selection of patients for this study followed a three-step process. In the first step, all patients with a possible diagnosis of thrombocytopenia or platelet disorder were identified in the database of the Thrombosis and Hemostasis Outpatient Clinic at HCFMUSP. This database consists of a spreadsheet containing the records of all patients who had consulted with the team from 1998 to 2023, regardless of the diagnosis; details are added to the database during the first consultation. A broad search in this database was performed to minimize losses, given the rarity of hereditary platelet disorders. Specific filters were used to select records labeled with terms such as "thrombocytopenia" or "platelet dysfunction" or "unexplained bleeding" or "storage pool disease" or "Glanzmann syndrome" or "Bernard-Soulier syndrome" or "May-Hegglin anomaly." Through this process, 857 patients were identified for further chart review.

The second step consisted of a chart review, in which the medical records of each patient identified in the first step were individually evaluated to determine whether the diagnosis was accurate. This was critical because the database used in the first step is updated only once, during the patient's first consultation, and the documented diagnosis is based on a preliminary assessment. As a result, diagnoses may change in future medical visits. A total of 319 patients identified in the first step did not have a digital medical record because they were lost to follow-up before the 2010 record migration, and their physical records were no longer available. Another 217 patients identified in the first step had only one or two medical visits recorded in their digital charts, also due to loss of follow-up. Without being able to confirm their diagnosis, these cases had to be excluded, which resulted in the retention of 321 patients.

In the third step, all charts were reviewed for confirmed or suspected diagnosis of an inherited platelet disorder. Of the 321 patients with available medical records, 261 patients were further excluded because their final diagnosis indicated a different condition, most commonly idiopathic thrombocytopenia, which accounted for approximately 50 % of these exclusions (130 cases). Other patients were excluded because of acquired causes of platelet disorders, including those related to medication, infection, hypersplenism, or thrombocytopenia that resolved during the follow-up (131 cases).

**Figure 1:**
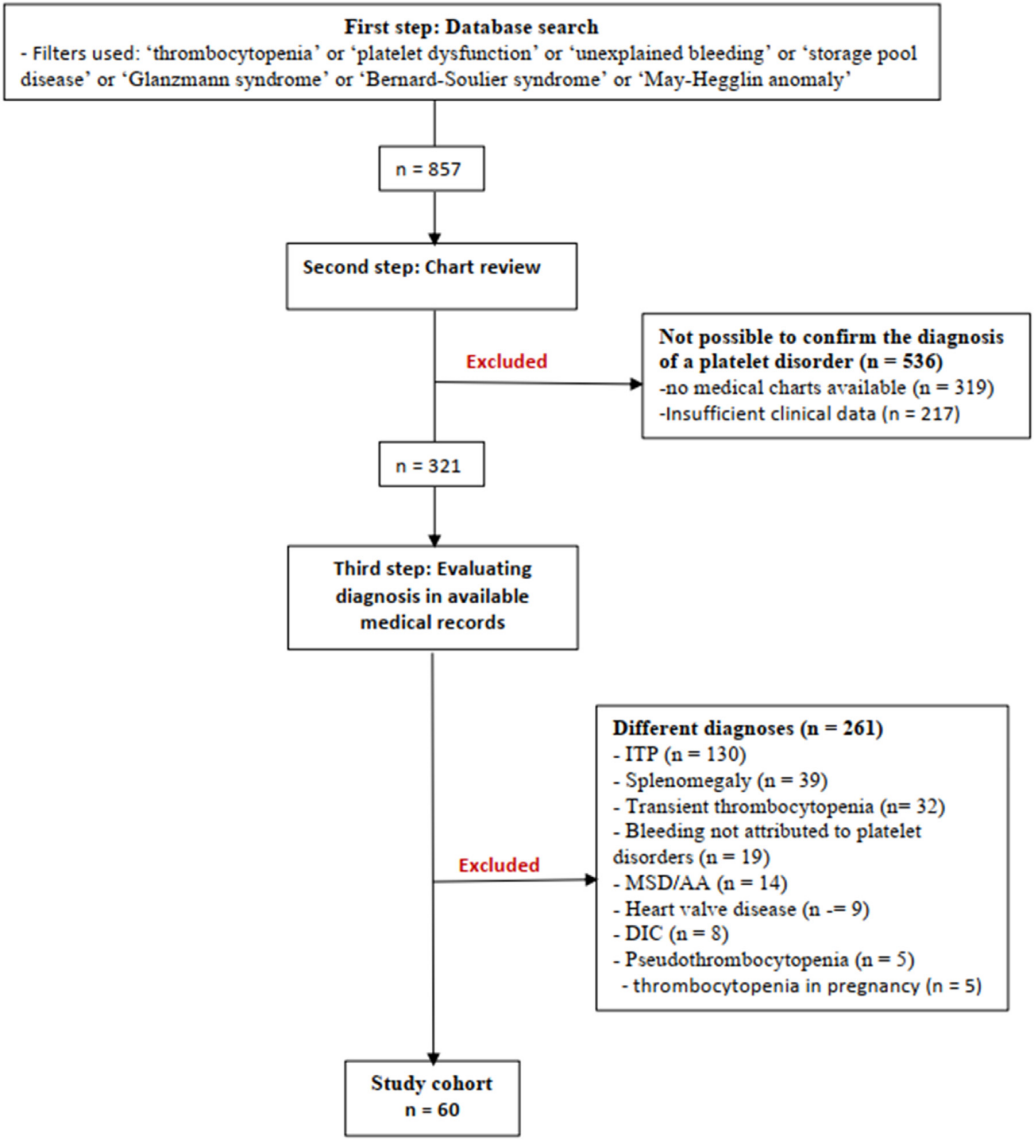
Flowchart of the study illustrating patient selection and reasons for exclusion. ITP: immune thrombocytopenia; MDS: Myelodysplastic syndrome; AA: aplastic anemia; DIC: disseminated intravascular coagulation.

Given the limited availability of certain diagnostic tests, such as electron microscopy and genetic mapping, the final cases were classified as suspected if patients had persistent thrombocytopenia since childhood without response to previous treatments, a suggestive family history, or increased bleeding associated with altered platelet aggregation and/or secretion tests. Figure 1 illustrates the study selection and reasons for exclusion.

This study was approved by the Research Ethics Committee of HCFMUSP (CAAE 67501923.7.0000.0068).

## Results

The final cohort comprised 60 patients, 39 (65 %) with IPFDs and 21 (35 %) with IPNDs. There was a predominance of women (75 %), with a median age of 48 years. The racial distribution was 42 % white, 42 % mixed-race, and 16 % black, without differences between the subgroups (Table1).

The suspicion of IPDs was based on factors obtained from the clinical history and laboratory tests. In cases where patients exhibited thrombocytopenia (13/21 - 62 %), IPNDs were suspected due to a positive family history of thrombocytopenia or after the diagnosis of immune thrombocytopenia (ITP) was excluded. A diagnosis of ITP was excluded based on the observation of long-standing, stable thrombocytopenia in the absence of a response to previous therapeutic interventions. In the IPND cohort, eight patients (38 %) received treatment for ITP without response, including corticosteroids (eight patients - 38 %), immunoglobulin (one patient - 5 %), immunosuppressants (one patient - 5 %) and splenectomy (two patients; - 10 %).

Eight patients (15 %) had previously received different diagnoses. One patient, initially suspected of having ITP, was confirmed to have MYH9-related thrombocytopenia, two cases initially diagnosed with ITP were confirmed as Bernard-Soulier syndrome, three cases of ITP were reclassified as unspecified inherited thrombocytopenia, and two patients initially suspected as having von Willebrand disease were reclassified as unspecified inherited platelet function disorder.

In the IPFD Group, the family history was positive in 20 out of 39 patients (51 %). Since some had concomitant thrombocytopenia, two patients received treatment for ITP (6 %), including corticosteroids (one patient - 3 %), immunoglobulin (one patient - 3 %), immunosuppressants (one patient - 3 %) and splenectomy (one patient - 3 %). In cases where patients exhibited excessive bleeding that was disproportionate to their platelet count, in addition to ruling out von Willebrand disease and other coagulopathies, platelet function was evaluated using aggregation tests, platelet immunophenotyping, and platelet secretion tests (lumiaggregometry). Abnormalities in these tests led to the suspicion or confirmation of IPFDs.

Nearly all patients (57 out of 60 patients - 95 %) faced some hemostatic challenge, including after dental extraction, or endoscopic or surgical procedures. Of a total of 51 women, 23 (51 %) had a previous pregnancy. There was a remarkable difference between the groups, with 12 out of 13 (92 %) IPND women reporting previous pregnancies, but only 11 out of 32 (34 %) IPFD women. The bleeding phenotype was assessed using the International Society on Thrombosis and Haemostasis Bleeding Assessment Tool (ISTH BAT) score^9^ giving a median score of 6 for the entire cohort (normal values are <4 for men and <6 for women). There was also a significant difference between subgroups: a median of 3.5 in the IPND Group and of 10 in the IPFD Group (Table 1).

**Table 1:** Clinical and laboratory features of the inherited platelet disorder cohort.

Variable	Total (*n* = 60)	IPND (*n* = 21)	IPFD (*n* = 39)
Female - n ( %)	45 (75)	13 (62)	32 (82)
Race - n ( %)			
White	25 (42)	8 (38)	17 (44)
Black	10 (16)	2 (10)	8 (20)
Mixed race	25 (42)	11 (52)	14 (36)
ISTH BAT score^a^ - median ± (IQR)	6.0 (3.0–11.0)	3.5 (1.8–4.3)	10.0 (6.0–13.0)
Highest platelet count during follow-up - x10^9^/L median ± (IQR)	178 (105–316)	108 (85–125)	279 (195–369)
Lowest platelet count during follow-up - x10^9^/L median ± (IQR)	90 (47–153)		
	56 (26–63)	132 (96–182)	
Highest MPV during follow-up (fL)^b^ - median ± (IQR)	11.5 (10.4–14.3)	14.1 (13.4–16.0)	11.1 (10.2–11.7)
Platelet aggregation test - n ( %)			
Patients with a platelet aggregation test result	34 (57)	9 (43)	25 (64)
Patients with abnormal platelet aggregation results	27 (79)	3 (33)	24 (96)
Consistent abnormalities^c^	19 (70)	2 (67)	17 (71)
Inconsistent abnormalities^c^	8 (30)	1 (33)	7 (29)
Platelet secretion assay - n ( %)			
Patients with platelet secretion assay result	16 (27)	2 (10)	14 (39)
Patients with abnormal results	7 (44)	1 (50)	6 (43)
Immunophenotyping test - n ( %)			
Patients with an immunophenotyping test result	27 (45)	6 (29)	21 (54)
Patients with abnormal immunophenotyping test results	15 (55)	0	15 (71)

IPND: inherited platelet number disorder; IPFD: inherited platelet function disorders; IQR: interquartile range; ISTH BAT: International Society on Thrombosis and Haemostasis Bleeding Assessment Tool; MPV: mean platelet volume.

a BAT score was available for 57 (95 %) IPD patients.

b The local MPV reference values range from 6.5 to 12.5 fl.

c For patients with more than one aggregation test, "consistent abnormalities" were considered when all the tests performed showed the same aggregation abnormality and "inconsistent abnormalities" when one or more results were discordant.

In the entire cohort, six patients (10 %) presented with associated syndromic abnormalities: three in the IPND Group (15 %) and three in the IPFD Group (8 %). The observed features included: two cases of deafness associated with nephropathy, one case of growth delay and heart disease, one case of cognitive impairment, and two cases of skeletal malformations with cognitive impairment.

In the IPND Group, the median highest and lowest platelet counts were 108 × 10^9^/L and 56 × 10^9^/L, respectively. As expected, the IPFD Group had normal or slightly reduced platelet counts, with median highest and lowest platelet counts of 279 × 10^9^/L and 132 × 10^9^/L, respectively. The median mean platelet volume (MPV) was 11.5 fL for the entire cohort; 14.1 fL for the IPND Group, and 11.1 fL for the IPFD Group (Table 1).

Platelet immunophenotyping was performed in 27 patients (45 %), six patients (29 %) in the IPND Group and 21 patients (54 %) in the IPFD Group. Of the total number of patients who underwent immunophenotyping, 15 (55 %) exhibited abnormal results, all of whom were in the IPFD Group (patients with diagnosis of Glanzmann thrombasthenia and Bernard-Soulier syndrome). Platelet aggregation tests were conducted in 34 cases (57 %), with nine (43 %) in the IPND Group and 25 (64 %) in the IPFD Group. Seven patients (21 %) had normal results and 27 (79 %) had altered platelet aggregation, however, discordant results were noted in eight (30 %) of the altered tests. Lastly, platelet secretion tests were performed in 16 patients (27 %), with two (10 %) in the IPND Group and 14 (39 %) in the IPFD Group (Table 1).

Based on the tests performed, the current cohort consists of 20 patients with confirmed diagnoses (33 %) and 40 with suspected diagnoses (67 %) of IPDs. Of the 20 confirmed diagnoses, one case in the IPND Group had a MYH9 macrothrombocytopenia confirmed by a genetic panel, and 19 in the IPFD Group had Glanzmann thrombasthenia (13 cases), Bernard-Soulier syndrome (five cases) and storage pool disease (one case) confirmed by electron microscopy. Of the 40 suspected diagnoses, 20 patients had unspecified IPNDs and 20 had unspecified IPFDs (Table 2).

**Table 2:** List of final diagnosis of the IPD cohort.

Diagnosis	
IPFD	n (%)
Storage pool disease	1 (2)
Glanzmann thrombasthenia	13 (22)
Bernard-Soulier syndrome	5 (8)
Unspecified inherited platelet function disorder^a^	20 (33)
IPND	n (%)
MYH9 macrothrombocytopenia	1 (2)
Unspecified inherited thrombocytopenia^b^	20 (33)

IPND: inherited platelet number disorder; IPFD: inherited platelet function disorders.

a Inherited platelet disorders were suspected, even in the absence of diagnostic testing, when the ISTH BAT score suggested a hemorrhagic phenotype and platelet aggregation or secretion was altered.

b inherited thrombocytopenia was suspected even when diagnostic tests were not available in cases of family history, altered platelet volume, persistent but stable long-term thrombocytopenia, and lack of response to immune thrombocytopenia treatment.

A total of 47 patients (78 %) received some treatment during follow-up; 43 % of the patients in the IPND Group and 97 % in the IPFD Group. The predominant treatments included the use of antifibrinolytics (73 %) and platelet transfusions (55 %) prior to surgical procedures or in cases of bleeding. In the IPFD Group, 90 % of patients received antifibrinolytic treatment and 77 % received platelet transfusions, while in the IPND Group, these numbers were 43 % and 14 %, respectively. Recombinant factor VIIa (FVIIa) was used in eight patients, all of whom had Glanzmann thrombasthenia.

## Discussion

Initially described in 1948, IPDs are a rare group of diseases, with few cohorts described in the literature. Most studies show no association of sex with diagnoses, but some indicate a higher prevalence of IPDs among women.^10^, ^11^, ^12^ In the current cohort, there was a predominance of women (75 %). A possible explanation is the greater frequency of hemostatic challenges that women face, such as menstruation and delivery, allowing increased bleeding tendencies to be identified. As for the distribution of ethnicity, this data is scarce in most publications. In the clinic of this study, there was a predominance of white and mixed-race patients with a smaller proportion of black patients. However, this ethnic distribution reflects the general Brazilian population.

The diagnosis of IPDs is challenging and probably most cases remain unidentified. Misdiagnoses are also common, often resulting in inadequate treatment.^13^^,^^14^ Among patients with IPNDs, up to 30 % receive an incorrect diagnosis of ITP and are sometimes treated with prolonged corticosteroid therapy, immunosuppressants, and even splenectomy.^1^^,^^15^

A similar occurrence was observed in this cohort with 38 % of patients in the IPND Group having received prior treatment for ITP, primarily corticosteroids (38 %), but also including splenectomy (10 %). The IPFD Group, which sometimes presents with thrombocytopenia, had a smaller proportion of patients receiving inappropriate treatments for ITP (6 %), including one splenectomy (3 %). When thrombocytopenia is present, it is often challenging to differentiate IPDs from ITP.^13^^,^^14^ This situation was found in the present cohort, as 10 % of the IPD patients were previously diagnosed with ITP. When increased bleeding occurs in the absence of thrombocytopenia, the main differential diagnosis was von Willebrand disease, with two changes in diagnosis during the follow-up.

A key factor in the investigation of IPDs is a family history of thrombocytopenia or increased bleeding, which was present in >50 % of both subgroups of the cohort. Similar data have been reported in other studies evaluating IPND,^13^^,^^16^ with a lack of data in IPFD cohorts. This study reinforces the importance of having this information in cases of diagnostic suspicion of IPD. The initial division into IPND and IPFD can also help with diagnosis, it distinguishes the primary differences between the subgroups and guides subsequent steps in the diagnostic process.

The IPFD Group exhibited a severe hemorrhagic phenotype with a median ISTH BAT score of 10. Conversely, the IPND Group showed a normal ISTH BAT score with a median of 3.5 with the caveat that we classified Bernard-Soulier syndrome and Glanzmann thrombasthenia as IPFD. These data are consistent with the literature, as in the ISTH BAT score validation study for the IPD population, the results showed a median of 9 for the IPFD Group and of 2 for the IPND Group.^17^ Comorbidities and syndromic features, although present and warranting evaluation, accounted for 10 % of the present sample. It is worth noting that only adult patients were evaluated in this study, and many syndromic patients often continue to be followed by pediatric and genetic teams.

As for laboratory propaedeutics, the platelet counts during follow-up were monitored, and thrombocytopenia was present in 25 % of patients with IPFD, however it was mild and disproportionate to the hemorrhagic phenotype. For the IPND Group, platelet size and morphology are important components of the diagnostic workup. In this cohort, 75 % of the IPND patients had altered MPV, in line with the association of these diseases with macro platelets and giant platelets, which is helpful for diagnosis.

Platelet aggregation was carried out on patients with no suspicion of Bernard-Soulier syndrome or Glanzmann's thrombasthenia, for whom direct immunophenotyping was chosen. Thus, of the 25 remaining cases of suspected IPFD, abnormal platelet aggregation consistently confirmed the diagnosis of platelet dysfunction in 17 (44 %). Secretion tests were altered in seven patients, detecting only one more whose aggregation test was inconsistent, but adding little information to the other six whose diagnosis was already confirmed with the aggregation test.

Mezanno et al. recommend the use of the platelet secretion assay as a first-line investigation for IPFD but highlight the lack of standardization as a major challenge to its implementation.^18^ In scenarios where resources are limited, the findings here call into question the role of platelet secretion tests. When aggregation is consistent, secretion can add cost with marginal benefits to diagnostic capacity. It therefore seems reasonable to focus on standardizing the platelet aggregation test and controlling pre-analytical factors. In the hospital of this study, access to genetic mapping tools and electron microscopy is lacking; the immature platelet fraction (IPF) is still being implemented and was not used for this study.

Some limitations of this study should be acknowledged. First, it was not possible to obtain data on platelet morphology from this cohort due to the lack of standardized reporting at the institution. Second, there were challenges in interpreting platelet aggregation results, as the assays exhibited a 30 % discordance rate, which compromised the diagnostic assessment. This variability was attributed to pre-analytical factors, which are frequently reported as sources of error in these assays. Third, most of the individuals identified in the first screening for patient selection had no further clinical data available due to loss of follow-up. In addition, many patients were excluded because of different diagnoses. This occurred because the approach to patient selection was highly inclusive, resulting in low specificity and giving rise to a large number of exclusions when the medical records were properly assessed. However, we believe that the loss of patients with IPD was minimal, as this is a rare, chronic, hereditary disease that generally requires continuous follow-up throughout life. Finally, we recognize that it is a challenge to diagnose IPDs without genetic evaluation, as two-thirds of our cohort still lack a confirmed diagnosis; however, this reflects the reality of most clinical services, particularly in low- and middle-income countries.

**Figure 2:**
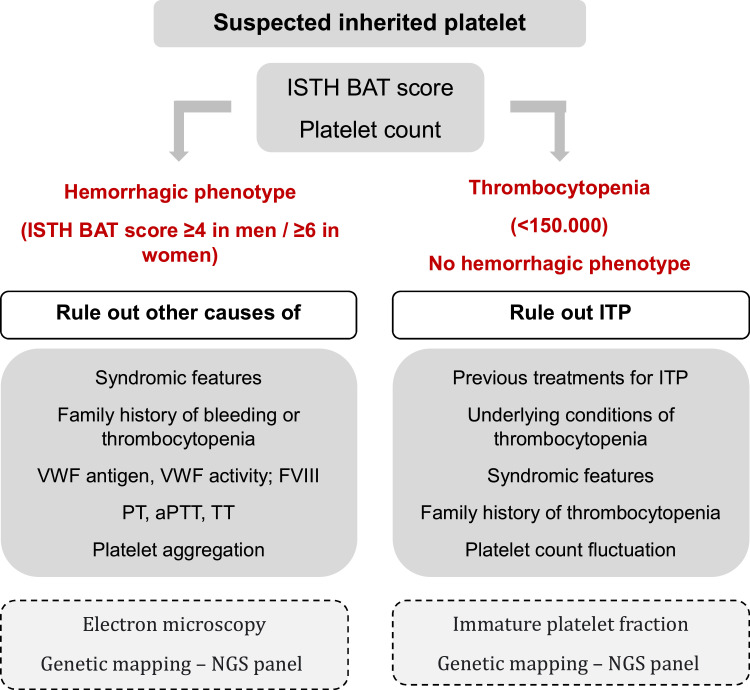
Institutional work-up for the diagnosis of inherited platelet disorders ISTH BAT: International Society on Thrombosis and Haemostasis bleeding assessment tool; ITP: immune thrombocytopenia; MPV: mean platelet volume; NGS panel: next-generation sequencing panel; VWF: von Willebrand factor; FVIII: factor VIII; PT: prothrombin time; aPTT: partial thromboplastin time; TT: thrombin time. Other possible tests that were not available in the institution at the time of this study have been highlighted in the dashed boxes.

Given the available resources, the most important points used in the investigation of IPDs are highlighted and summarized in Figure 2, as an example of a diagnostic flow chart for these conditions.

Regarding management, the IPFD Group undergoes more therapeutic interventions, possibly due to a higher bleeding tendency,^19^ similar to what was observed in this cohort. Platelet transfusions and the use of antifibrinolytics were frequent in this Group. On the other hand, IPNDs were treated less frequently, with transfusion triggers depending on the platelet count.

## Conclusion

Patients with IPDs may have a higher risk of bleeding, and the vast majority will be exposed to some hemostatic challenge during their lives. Identifying the correct condition makes it possible to provide appropriate treatment and follow-up. In scenarios with limited resources, confirmation of the diagnosis may not always be possible. Nevertheless, the documentation of critical elements of personal and family history, the assessment of bleeding risk, and the use of quantitative and qualitative platelet tests facilitate the identification of patients.

The present cohort serves as an example of the management of IPDs within a public healthcare system, devoid of genetic mapping exams. Moreover, the description of this cohort sheds light on the unique characteristics of these disorders within a Latin-American population.

## Conflicts of interest

The authors declare that the research was conducted in the absence of any commercial or financial relationships that could be construed as a potential conflict of interest.
